# Use of the Certificate for Pharmaceutical Products (CPP) in 18 Maturing Pharmaceutical Markets: Comparing Agency Guidelines with Company Practice

**DOI:** 10.1007/s43441-020-00196-2

**Published:** 2020-07-02

**Authors:** Céline Rodier, Magda Bujar, Neil McAuslane, Prisha Patel, Lawrence Liberti

**Affiliations:** 1 Centre for Innovation in Regulatory Science (CIRS), London, UK; 2 Friars House, 160 Blackfriars Road, London, SE1 8EZ UK

**Keywords:** Certificate of pharmaceutical product, CPP, CPP timing, CPP recommendations

## Abstract

**Background:**

The certificate of pharmaceutical product (CPP) was implemented to accelerate the availability of new drugs in developing countries by providing evidence of the quality of products and reducing the time to market through reliance on a prior trusted analysis. However, the CPP format, issuing process and use have not been revised since 1997 and there are significant differences among countries in regard to requirements for CPP timing, terminology, and format. We sought to determine current CPP practices versus national regulatory guidelines and to inform recommendations for the efficient use of the CPP based on the needs of the modern regulatory environment.

**Methods:**

We conducted a comparative analysis of company practice versus agency guidelines across 18 maturing pharmaceutical markets using data from the Cortellis for Regulatory Intelligence® (CRI) and the Centre for Innovation in Regulatory Science (CIRS) Emerging Markets Regulatory Review Times (EMaRReT) databases and regulatory authorities’ websites.

**Results:**

Of the studied 18 countries, 16 require the CPP for submission of new registrations; many accept alternative documentation but most still require legalization of the CPP and many are not compliant with the complex CPP format. Additional complicating factors include language requirements and varying local guidelines for CPP submission timing and validity dates.

**Conclusions:**

With the implementation of a number of suggested improvements, the CPP can continue to serve an important role in streamlining regulatory efficiency and provide confidence in new medicines, ensuring a more efficient and effective approval process and expediting patient access to safe and effective medicines worldwide.

## Introduction

The World Health Organization (WHO) Certification Scheme was initially implemented to accelerate the availability of new drugs in developing countries by providing evidence of the quality of products through the use of the Certificate of Pharmaceutical Product (CPP) [1, 2]. The Certification Scheme has been in operation since 1969 and was amended in 1975, 1988, 1992, and 1997 [3–7].

The use of the CPP was expected to benefit all parties, including regulatory agencies, patients, and pharmaceutical companies, improving the internationalization of product availability and reducing the time to market by limiting duplicative assessments through reliance on a prior trusted analysis [8, 9]. In April 2018 the WHO issued a draft proposal for the revision of the Scheme on the quality of pharmaceutical products moving in international commerce [10].

It is expected that rational use of the CPP promotes simplification and convergence of practices to enhance the globalization of the pharmaceutical market, regulatory environment, and product life cycle management. By relying on the previous thorough evaluation of the quality, safety, and efficacy of a product, regulatory staff in maturing agencies can provide added-value rather than duplicative assessment activities [8, 11]. However, the global economic and regulatory environment have changed significantly in recent years, with the introduction of new technologies for example, leading to increased complexity in the expectations and review practices of national regulatory authority (NRAs), with more detailed data requirements and increasingly sophisticated local assessments that may exceed or deviate from international expectations. For example, while a number of innovative products are given a marketing authorization by the FDA or EMA based on phase 2 data, other countries such as Colombia generally require more mature clinical data to support their regulatory decision. Consequently, patient access to medicines may be delayed by a variety of factors such as additional national requirements not included in the scheme (for example, its legislation and translation), communication between issuing and recipient authorities, and in part because the CPP is still required by many NRAs at the time of submission [2, 12]. Also, the CPP format, issuing process, and use have not been revised since 1997 [13].

Through a literature review, we identified other issues that hinder efficient use of the CPP, including the fact that some NRAs need more than one CPP and that there is only now is there a move towards internationally harmonized definitions of CPP-related terminology, such as *issuing authority*, *country of origin (COO),* or *reference country*. Resource constraints in issuing authorities may affect the time to issue a CPP [2]. Also, timing for CPP submission remains nationally determined [2, 14]; some NRAs do not issue the CPP in the WHO format [15]; some importing countries require that the product be on sale in the issuing country [12] and there may be product differences between the recipient and issuing agency [2]. Finally, because a CPP may be subject to fraud and counterfeiting [13] some NRAs require legalization of the document [2, 10].

Recent national and regional initiatives to assess CPP requirements have taken place. In 2018, the Pan American Health Organization (PAHO)/ Pan American Network Drug Regulatory Harmonization (PANDRH)/WHO initiative assessed CPP requirements for drug registration processes in the Americas. Data were collected from 41 NRAs and companies, followed by a structured discussion on CPP-related practices and requirements [16]. Results show that the majority of agencies studied requested the CPP to be notarized, authenticated, or legalized and that no clear timelines were established for CPP issuance after a request was submitted. A previous review of CPP requirements in Latin America (LATAM) was conducted in 2013 and showed that most of countries studied in the region would not accept an application without a CPP [17]. The need for CPP documentation and timing was also discussed at the 2018 International Conference of Drug Regulatory Authorities (ICDRA) meeting and the results of the 2018 WHO consultation on changes to the Certification Scheme were presented at the 53rd meeting of the WHO Expert Committee on Specifications for Pharmaceutical Preparations (ECSPP).

We hypothesize that the role of the CPP, as defined by individual national regulatory statutes, may differ from the ways that the CPP is applied in practice, resulting in an impact in regulatory efficiency. Therefore, we conducted a comparative analysis of company practice versus agencies guidelines across 18 maturing pharmaceutical markets to determine current practices and to inform recommendations for the efficient use of the CPP.

## Methods

### Scope

Information was collected regarding the use of the CPP by the following 18 countries across three regions:

- LATAM: Argentina, Brazil, Colombia, Mexico.
- Europe, Middle East and Africa: Algeria, Egypt, Israel, Saudi Arabia, South Africa, Russia, Turkey.
- Asia Pacific (ASIA): India, China, Indonesia, Malaysia, Singapore, South Korea, Taiwan.

These countries were selected in 2007, when the Centre for Innovation in Regulatory Science (CIRS) established the Emerging Markets Regulatory Review Times (EMaRReT) database through member company identification of countries of commercial interest, data for which would help with global development strategy.

### Data

#### Data Sources

##### CPP Use Based on Agency Practice

The Clarivate Analytics Cortellis for Regulatory Intelligence® (CRI) database was used to extract CPP information for each target country. CRI is a single, comprehensive source for global regulatory information on the development of drugs, biologics, medical devices, and in vitro diagnostic devices across the product life cycle, produced by Clarivate Analytics. The findings were collected during April 2018 and complemented with documentation available from NRA websites.

##### CPP Use Based on Company Practice

Data for company practice were derived by an analysis of the EMaRReT database, maintained by CIRS. EMaRReT provides regulatory benchmarking and trend analysis of regulatory approval times for 18 Emerging Market countries [18].

The data collected during early 2018 and used for these analyses include those for all new active substances (NASs) approved between 2016 and 2017 in the 18 countries from 12 multinational companies. An NAS in our study is the active substance that is intended to furnish pharmacological activity or other direct effect to a pharmaceutical product. This may be a chemical, biological, biotechnology, or radiopharmaceutical substance that is or is destined to be made available as a prescription-only medicine, to be used for the cure, alleviation, treatment, prevention, or in vivo diagnosis of diseases in humans. Under the *biological* definition, we include a substance isolated from animal tissues, such as vaccines, hormones and antigens; or plant alkaloids.

##### Data Extraction

The following information was extracted from both agency guidelines and the EMaRReT database:

- CPP requirement for NASs.
- Need for CPP legalization.
- CPP-issuing country.
- CPP submission timing.
- Alternative document submitted instead of CPP.

## Results

### CPP requirement for NASs

#### Agency Information and Guidelines

**Figure 1.**
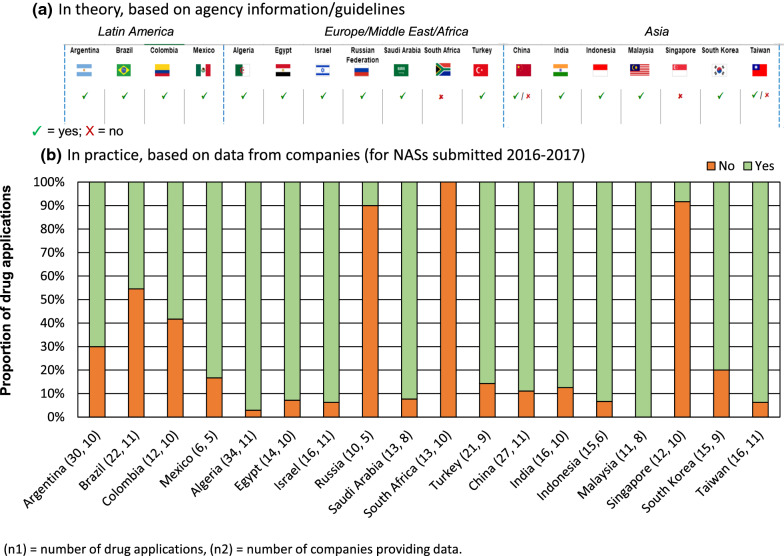
Requirement for a Certificate of Pharmaceutical Product for New Active Substances (NASs) Based on **a** Agency Information and **b** Company Practice.

Most agencies, 78% (14 of 18), required a CPP for all NAS registrations (Fig. 1a). For Singapore and South Africa, the CPP was not required but could be submitted. For China and Taiwan, the CPP depended on the route. More specifically, in China, the CPP was not mandatory for new drug applications if the company submitted data from a multinational clinical trial. In Taiwan, the CPP was requested if no clinical studies had been conducted in the country. For 89% of the NRAs that required a CPP, the document had to follow the WHO format; exceptions were China and Mexico. In Mexico, the CPP was not required for NASs that had not been commercialized in any other country and the CPP could be replaced by a Clinical Research Report where Mexican patients participated in the clinical trial [19].

Our study shows that though English is the most commonly used language for issuing the CPP, it is in combination (four countries) or not (ten countries) with local languages in 78% (14 of 18) of the countries.

#### Company Experience

The practice of companies in this cohort for NASs submitted in 2016–2017 was generally in line with that dictated by NRA guidelines (Fig. 1b). Nevertheless, company experience showed flexibility, even among agencies requiring a CPP.

Based on data from 11 companies, 55% of NASs (12 of 22) approved in Brazil did not require a CPP, despite agency information implying otherwise [20]; alternative evidence may have been negotiated. This was also observed for Russia, with 90% (9 of 10) applications from five companies not requiring a CPP. Alternative evidence was provided such as the Free Sales Certificate (FSC) or approval letter from reference agencies. A Free Sales Certificate is a document required in certain countries or for certain commodities (such as pharmaceuticals), certifying that the specified imported goods are normally and freely sold in the exporting country's open markets and are approved for export.

Reviewing agency guidelines, we observed that 78% of NRAs (14 of 18) required the CPP as a component of the NAS registration process, but when the CPP was not readily available, 64% (9 of 14) of NRAs (Colombia, Egypt, India, Indonesia, Israel, Russian Federation, Saudi Arabia, Singapore, South Africa) accepted the FSC as an alternative.

### Need for CPP Legalization

#### Agency Information and Guidelines

**Figure 2.**
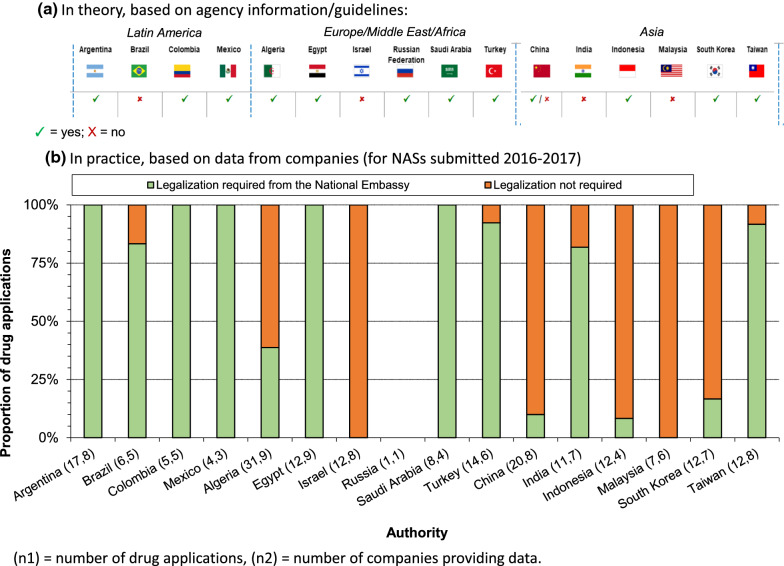
Requirement for a Certificate of Pharmaceutical Product Legalization Based on **a** Agency Information and **b** Company Practice.

For a majority of the NRAs requiring a CPP, 69% (11 of 16) required the CPP, original or a copy, to be legalized by the national embassy of the country where the document was to be presented. Four countries (Brazil, India, Israel, Malaysia) did not require this legalization, and China required legalization of copies but not of the original CPP (Fig. 2a).

#### Company Experience

Legalization practices differed across agencies. Indeed, in Brazil and India, guidelines stated that CPP legalization was not required, but was required in practice for the majority of the applications. Specifically, 5 of 6 applications in Brazil and 9 of 11 applications in India in our study required legalization. Although certain agencies may not require a legalized CPP, they may expect it to be apostilled (a form of authentication for documents that will be used in countries that participate in the Hague Convention), or notarized, or for the translation (as opposed to original document) to be legalized. A reverse situation was observed in Indonesia and South Korea, where legalization was required by guidelines, but company practice showed 11 of 12 and 10 of 12 applications, respectively, not requiring this step (Fig. 2b).

### Issuing Country

Despite initiatives to harmonize regulatory practices, we found the concept of COO to be defined differently by most agencies. For 56% of NRAs (9 of 16); China, India, Indonesia, Malaysia, Mexico, Russian Federation, Saudi Arabia, South Korea, Taiwan), COO was defined as the country where the product was manufactured. In other cases, COO related to the country where the marketing authorization holder or the company headquarter was located (eight countries; Algeria, Argentina, Brazil, China, Colombia, Egypt, Saudi Arabia, Turkey). Additionally, for Indonesia, the COO could be the CPP-issuing country and for Egypt the drug-exporting country. It was also possible for a country to have multiple definitions; for example, China can consider the COO as coming from the country where the finished product is, in order, manufactured (first), the country of the MAH (second) and the country of company’s headquarters (third).

There was also a divergence regarding which authority should issue the CPP, where for 80% countries (12 of 15; Algeria, Brazil, China, Colombia, Egypt, India, Malaysia, Mexico, Saudi Arabia, South Korea, Taiwan, Turkey), the CPP could be issued by the COO. In other cases, it had to be a recognized health authority or the relevant country of authorization of the medicine. Singapore, Taiwan, and Russia were removed from this particular analysis because of insufficient company-supplied product data in the EMaRRET database.

According to company practice, the majority of the companies had the CPP issued from the COO, as per the agency requirements 67% (12 of 18) agencies required CPP from COO in accordance to their guidelines. Nevertheless, for certain NRAs there seemed to be flexibilities, such as in India, where 50% of NASs were issued from a non-COO country.

Based on agency guidelines, the US Food and Drug Administration (FDA) and European Medicines Agency (EMA) were the most commonly recognized CPP-issuing health authorities, followed by Health Canada, Japan PMDA, Swissmedic, and the United Kingdom MHRA. This was in line with data from companies, showing that CPPs were issued from the USA for 66% and from the EU for 25% of 173 NASs submitted in 2016–2017; other CPP-issuing countries included Switzerland, UK and Denmark. Importantly, lead times for national agencies to issue a CPP based on their guidelines could vary widely. For the EMA it was within ten working days (standard procedure) or two working days (urgent procedure) [20]; for the US FDA, within 20 days [21]. Six countries indicated a lead time of two weeks, and timing ranged from 1 day in Colombia to 9 months in India.

### Timing for CPP Submission

**Figure 3.**
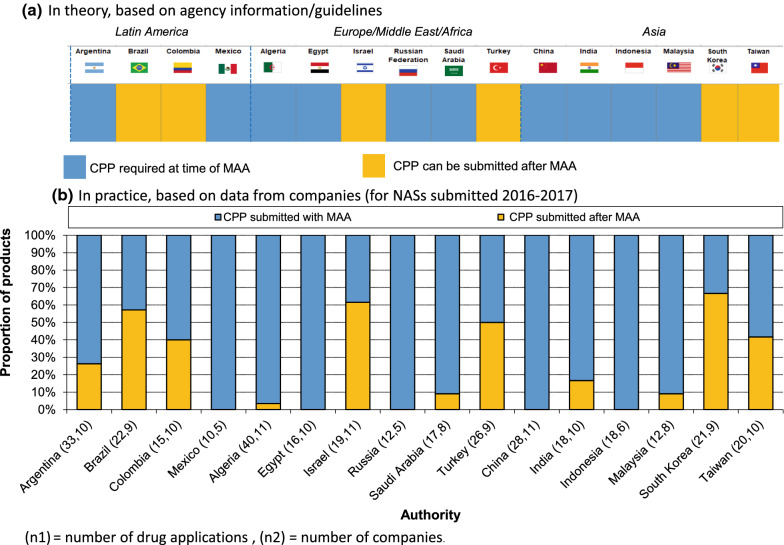
Certificate of Pharmaceutical Product (CPP) Submission Timing, Relative to New Active Substance (NAS) Marketing Authorization Application (MAA), Based on **a** Agency Information and **b** Company Practice.

For the majority of the NRAs (63%; 10 of 16), the CPP should be provided at the time of submission of the NAS marketing authorization application (MAA), whereas in Brazil, Colombia, Israel, Turkey, South Korea, and Taiwan, the CPP could be submitted later, but prior to obtaining regulatory approval (Fig. 3a). Indeed, according to company-provided data, there was flexibility in the CPP submission process for those agencies, although the CPP was still sometimes submitted at the time of MAA submission (Fig. 3b). In Argentina, approximately 25% of CPPs were submitted post-submission.

### Effect of CPP on Product Rollout

**Figure 4.**
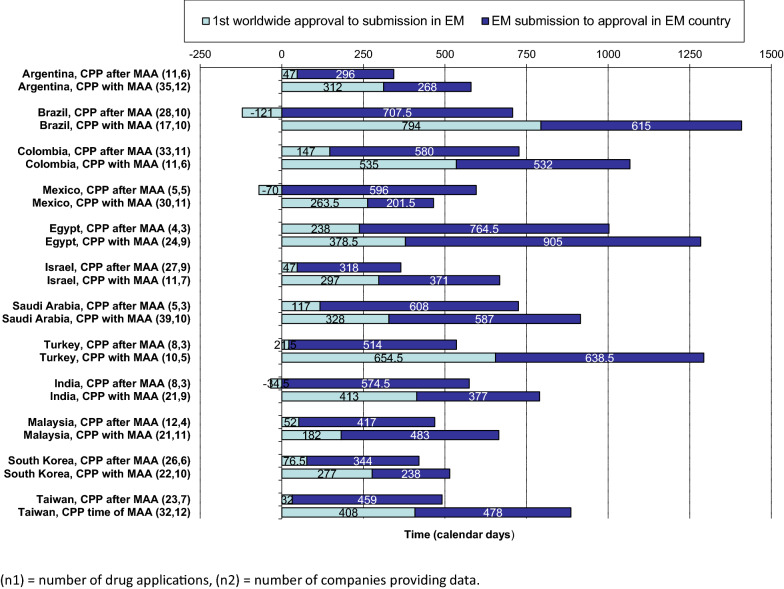
Submitting a Certificate of Pharmaceutical Product (CPP) at the Time of a New Active Substance (NAS) Marketing Authorization Application (MAA) or Afterward: the Effect on NAS Rollout in Emerging Markets.

The effect of the CPP submission timing on product rollout (the time from the first-in-world agency approval to the agency approval in a maturing country) was assessed in 12 of the jurisdictions (where there were sufficient data). (Fig. 4). Where the CPP was submitted after the marketing authorization application (MAA), the overall timeline from first-in-world approval to emerging market approval was shorter for 11 of 12 authorities (Mexico being the exception) compared with when the CPP was submitted at the time of MAA (despite longer approval times in certain countries such as Turkey and Egypt). Across all 12 authorities, the overall median from first-in-world approval to emerging market approval was 477 days where CPP was submitted after MAA, compared to 965 days where CPP was submitted at the time of MAA. Indeed, where CPP was submitted after MAA, the faster roll out was driven primarily by shortening of the time from first-in-world approval to submission to EM an emerging market NRA. In certain cases, particularly Brazil and Mexico, submitting the CPP post-MAA also resulted in the median submission occurring prior to first-in-world approval (121 and 70 days, respectively) In certain cases, particularly Brazil and Mexico, submitting the CPP after filing the application (but before approval) resulted in the submission occurring prior to the first-in-world approval (median of 121 and 70 days, respectively).

## Discussion

The main reason for the CPP revisions in 1992 and 1997 was to reduce the time to submit medicines in maturing markets. Since the last revision, there has been a number of change recommendations to make the CPP more relevant to the evolving development of globalized medicines [10, 16], as discussed below.

This study evaluated both formal agency guidelines and actual company CPP use across 18 maturing markets and our observations highlighted the need for CPP refinements at both the country and WHO level [10, 16]. Through our literature search we have identified several recommendations for change to the CPP since the last update of the Scheme, as discussed below.

### CPP Requirement and Timing for NASs

Not all maturing agencies studied always required a CPP as a component of an MAA. Singapore and South Africa did not require it, and in China, Taiwan, and Mexico, it was dependent on product type or the conduct of local clinical trials.

Our observation was that the practice of companies was generally aligned with agencies’ guidelines. However, companies were able to submit the CPP at the time of approval across a number of countries, suggesting flexibility in the process.

As discussed by Withing, a more rational use of the CPP and its timing would facilitate earlier submissions [2]. Despite an increasing flexibility in timing, most NRAs studied (63%) required the CPP at the time of MAA. That almost 40% of agencies are flexible in this approach may reflect their desire to accommodate the sometimes onerous and lengthy process required to obtain a CPP. It could also be a reflection of the way the agencies uses the CPP; for example, not to replace part of their review, but rather to confirm their decision before approval.

Encouraging the submission of an MAA before the physical availability of a CPP can contribute to regulatory efficiency and reduced overall rollout timelines as observed, for example, in Brazil and Mexico. Indeed, CPP submission post-MAA/pre-approval shortens the overall rollout, compared with CPP submission with the MAA [12]. Further, we agree with the observation that where the agency is conducting a full assessment of quality, pre-clinical and clinical data, a CPP should not be necessary to guarantee these attributes and only adds time to the regulatory process [15, 22].

Consequently, we support the position of the International Federation of Pharmaceutical Manufacturers and Associations (IFPMA) [15] to adhere to the original concept of the WHO Scheme and use the CPP according to its intended purpose. The CPP-requesting NRAs should adapt their acceptance timing requirements according to the assessment process they are conducting. Provision of the CPP during the review process should permit either an accelerated full review process, or an abbreviated reliance/recognition procedure based on an assessment already conducted by a WHO-listed certifying authority or other recognized reference agency. Additionally, independently of where the product is manufactured, released, and exported, a CPP should be available for issue as soon as the product is approved by the certifying authority.

### Waiving the Need for CPP Legalization

CPP legalization is not a WHO requirement [22]. In Brazil, India, Israel, South Africa, and Malaysia and China (for original documents) guidelines indicate that it is not required. The WHO has suggested that legalization is not aligned with the Scheme purpose, the CPP already being a legal document [22]. However, 11 of the NRAs in scope still require legalization, although company practice suggests flexibility. Legalization was found in one study to not add value in ensuring the quality of a product and increased both the time and cost of the regulatory process [8].

Agencies have cited a concern regarding the potential for counterfeit CPPs without legalization, and legalization that can be faked [2, 13]. However, there are simple alternatives to guarantee CPP authenticity such as an electronic CPP [23] or the use of publicly available approval documentation such as European Public Assessment Reports. Final recommendations made at the 2018 WHO ICDRA meeting stated that “WHO should advocate for the use of an electronic CPP template by issuing and receiving authorities to expedite the process and mitigate against any further need for legalization” [16]. Nonetheless, although an electronic format allows faster communication [23] and is a forward-thinking recommendation that we support, its implementation may be slowed as this will require an investment in infrastructure for agencies that may already be struggling with resource constraints”.

### Issuing Country

A significant point of variability across the reviewed NRAs in CPP use centers on the definition of “country of origin”, encompassing the country of manufacture, country of authorization, and country of headquarters, among others. Such variety is mainly related to the increased complexity of the global supply chain and various regulatory frameworks in place [14]. Aligning upon a standard definition would provide more consistency and predictability for sponsors and for CPP-issuing agencies. As the WHO develops its policy for qualifying WHO-listed authorities, based on their Global Benchmarking Tool maturity level, this will help receiving agencies to have confidence in the information supplied in the CPP. CPPs should not be issued by NRAs not qualified by the WHO as a CPP-issuing agency The CPP is based on the assumption that the authorities issuing a CPP have the capacity to assess the quality, safety, and efficacy of the product they approve for marketing. Therefore, we recommend that CPPs should not be issued by NRAs not qualified by WHO as a CPP-issuing agency [24]. Additionally, the CPP should be updated to reflect changes in which the issuing country is no longer the country of product manufacture. Final recommendations made at the 2018 WHO ICDRA meeting state that “The CPP template should be updated to reflect current manufacturing situations by including: (a) the sites of manufacture with addresses, and (b) a reminder that the receiving country should check that the product being shipped to it is exactly the same as the product being certified by the issuing country” [16]. Challenges occur when an agency requires information that the CPP-issuing agency has not provided and which cannot be obtained. Therefore, a coordinated implementation of CPP changes is needed across agencies.

### Alternative Evidence

We observed that a number of NRAs accept alternative documents to the CPP; however, applicants may be confused by the options for different documents and the lack of consistency may contribute to increased complexity.

### Reliance Approach

As the WHO and others advocate for agencies to consider establishing formal reliance pathways as an alternative to a full dossier review, it is important that any changes to the CPP will enable agencies to use such pathways efficiently [25, 26]. Final 2018 ICDRA recommendations included that “the WHO should advocate for the CPP standard procedure, specifying that value-added, unredacted documents either accompany the CPP or are provided upon request by any receiving agency” [16].

Our study has shown the continuing importance of the CPP in the approval of NASs (89%; 16 of 18 NRAs requiring this document). However, several factors complicate the CPP process today: most importing countries still require legalization of the CPP; the required WHO format for CPPs is complex and may represent challenges for compliance; [27] the language requirement for the document; identifying the appropriate reference agency issuing the CPP; local variety of CPP validity and expiry dates; and requests for additional information beyond the standard CPP requirements.

These observations are particularly relevant in light of the increasing use of reliance pathways by maturing agencies: most NRAs that have implemented verification and /or abridged NAS procedures are CPP dependent, which means the availability of medicines must wait for prior approvals and documentation [28]. Among the agencies applying formal reliance pathways, only Singapore did not require a CPP, but rather an official approval letter from two reference agencies, or an equivalent document that certifies the registration status of the drug product.

Chong and associates et al. proposed key performance indicators to measure regulatory convergence and cooperation in the Asia Pacific Economic Co-operations countries (APEC) by 2020, such as the number of economies establishing reliance or mutual recognition; removing CPP dependence; aligning with or implementing International Council for Harmonisation of Technical Requirements for Pharmaceuticals for Human Use (ICH) guidelines; and complying with standards such as the Pharmaceutical Inspection Cooperation Scheme (PIC/S) [29]. They recommended that the CPP continues to have a role depending on the APEC country reliance system, whenever relevant and as long as it remains updated:

1. CPP to be used in lieu of full review for agencies that have very limited resources and yet to mature (not a member of PIC/S)
2. Public assessment report to be used for agencies that are members of PIC/S and are conducting full review independently to learn the review/approval of product based on ICH in preparation of work-sharing/joint review in the near future with like-minded agency.

Therefore, as NRAs embed formal reliance processes, it will be of interest to observe how the role of the CPP will change.

## Limitations of study

CIRS observations were derived from published guidelines and some source documents were not comprehensive and therefore were subject to interpretation by the authors. Industry experience was focused on NASs MAAs submitted by a representative cohort of 12 multinational companies, but did not reflect all NASs reviewed by all agencies during this time period.

## Conclusions

The WHO 2018 draft proposal to update the use of the CPP [10] is a major step in reflecting the current use, value and role of the CPP in regulatory assessment in the global context. In the proper format, with timely availability, and as a supportive decision tool for reliance reviews, the CPP can continue to serve an important role to streamline regulatory efficiency and provide confidence in the quality, safety and efficacy of products approved by WHO-listed agencies. We observed that approval timelines can be improved by the effective and flexible use of the CPP, which expedites patient access to safe and effective medicines, especially if MAAs are made before the CPP become available. In addition, our data find that a flexible approach by NRAs to the use of the CPP facilitates MAA. Therefore, we are supportive of the implementation of a simplified globally harmonized CPP process, which will require close interactions between the WHO, issuing and requesting agencies, and industry stakeholders. The WHO 2018 draft proposal to update the use of the CPP [10] is a major step in reflecting the current use, value and role of the CPP in regulatory assessment in the global context. In the proper format, with timely availability, and as a supportive decision tool for reliance reviews, the CPP can continue to serve an important role to streamline regulatory efficiency and provide confidence in the quality, safety and efficacy of products approved by WHO-listed agencies.

The WHO 2018 draft proposal and ICDRA 2018 meeting recommendations, combined with our findings, suggest it is appropriate to address potential changes to the WHO Scheme with a more flexible use of CPP, as custom and practice tend to adapt faster than guidelines [16].

Ultimately, the goal of the CPP is to support the added-value work of health authorities, particularly in maturing countries. The implementation of a simplified globally harmonized CPP process will require close interactions between the WHO, issuing and requesting agencies, and industry stakeholders. Although there are a number of challenges to be addressed, it is important to make ensure that the CPP Scheme remains fit-for-purpose. Accordingly, a number of suggestions have been made herein and the outcome of the suggested improvements could promote a more efficient and effective approval process, that will improve accelerate product approval timelines and expedite patient access to safe and effective medicines worldwide.
